# Possible Influence of Weight Gain and Creatinine Levels in Predicting Response to Nivolumab: A Multicenter Analysis

**DOI:** 10.3390/metabo10120510

**Published:** 2020-12-14

**Authors:** Cornelia Nitipir, Cristina Orlov-Slavu, Lucian Alecu, Iulian Slavu, Anca Pantea-Stoian, Ionela Daniela Celmare, Mihaela Olaru, Valentin Calu, Andra-Iulia Suceveanu, Laura Mazilu, Andreea-Daniela Gheorghe, Adelina Silviana Gheorghe, Catalina Poiana, Razvan Hainarosie, Sanziana Octavia Ionescu, Dana Lucia Stanculeanu

**Affiliations:** 1 Clinic of Medical Oncology, Elias University Emergency Hospital, 11468 Bucharest, Romania; nitipir2003@gmail.com (C.N.); orlov.cristina@gmail.com (C.O.-S.); celmaredana1@gmail.com (I.D.C.); miha0611@yahoo.com (M.O.); 2 Faculty of Medicine, Carol Davila University of Medicine and Pharmacy, 020021 Bucharest, Romania; razvan@riaclinic.com (R.H.); dlstanculeanu@gmail.com (D.L.S.); 3 Clinic of General Surgery, Agrippa Ionescu Emergency Hospital, 011365 Bucharest, Romania; iulian.slavu@yahoo.com; 4 Department of Diabetes, Nutrition and Metabolic Diseases, Carol Davila University of Medicine and Pharmacy, 020021 Bucharest, Romania; ancastoian@yahoo.com; 5 Clinic of General Surgery, Elias University Emergency Hospital, 11468 Bucharest, Romania; drcalu@yahoo.com; 6 Department of Gastroenterology, Ovidius University of Constanta, Clinical Emergency Hospital of Constanta, 900591 Constanta, Romania; andrasuceveanu@yahoo.com; 7 Department of Oncology, Ovidius University of Constanta, Clinical Emergency Hospital of Constanta, 900591 Constanta, Romania; andreea_gheorghe78@yahoo.com; 8 Clinic of Oncology, Bucharest Institute of Oncology ‘Al Trestioreanu’, 022328 Bucharest, Romania; adelina.silvana.gheorghe@gmail.com; 9 Clinic of Endocrinology, National Institute of Endocrinology ‘C.I.Parhon’, 011863 Bucharest, Romania; endoparhon@gmail.com; 10 Institiute of Phonoaudiology and Functional ENT Surgery—Prof Dr Dorin Hociota, 061344 Bucharest, Romania; 11 Clinic of Surgical Oncology, Bucharest Institute of Oncology ‘Al Trestioreanu’, 022328 Bucharest, Romania; secretariat@iob.ro

**Keywords:** prognostic, body mass index, sarcopenia

## Abstract

Literature suggests that high body mass index can be correlated with better response to immune checkpoint inhibitors. On the other hand, sarcopenia seems to be a negative predictive marker. The present analysis is a retrospective, multicenter trial that included patients with metastatic melanoma, non-small cell lung cancer (NSCLC), and renal cell carcinoma treated with nivolumab between 2018 and 2020. Patients were stratified by creatinine levels both at treatment initiation and at first follow-up (at three months) and by BMI for the same intervals, as recorded in the patients’ charts. Creatinine was considered a surrogate marker for sarcopenia. IBM SPSS version 20 was used for statistical analysis. A total of 57 (n = 57) patients were included in the trial. Overall response rate (ORR) for the entire population was 38.59% (*p* = 0.02). Patients with BMI lower than 25 had an ORR of 28.5% (*p* = 0.003), whereas patients with BMI higher than 25 had an ORR of 42.3% (*p* = 0.002). Patients who gained weight during treatment had a lower probability of having progressive disease (OR = 0.4 [95% CI; 0.4–1.2]), as did patients with creatinine higher than 0.9 (OR = 0.39 [95% CI: 0.13–1.14]). No superiority was found in progression-free survival (PFS) when patients were dichotomized for BMI = 25 or BMI = 18.5. Mean PFS in the BMI under 18.5 group was 10.2 months [95% CI: 5.8–23.1], versus 11.2 for BMI over 18.5 [95% CI: 5.3–25.3], *p* < 0.03. Mean PFS for the BMI under 25 was 11.2 months [95% CI: 7.2–20.1], vs. 13.3 months [95% CI: 6.4–22] for the BMI over 25, *p* < 0.001. There were also differences in PFS in the patients with baseline creatinine over 0.9 when compared with under 0.9 values. Mean PFS in the first group was 19.78 months [95% CI: 16.23–22.9] vs. 16.1 [95% CI: 12.2–20.3], *p* < 0.001. Patients treated with nivolumab who have weight gain during treatment have a better PFS than the ones who do not. Creatinine levels of over 0.9 at treatment initiation also have positive predictive value.

## 1. Introduction

Nivolumab, an anti-Programmed-Death-1 (PD-1) antibody has brought considerable benefit in the treatment of melanoma, non-small cell lung cancer (NSCLC), and renal cell carcinoma. Selecting patients for this treatment is still a challenge. The most studied predictive biomarkers for response to checkpoint inhibitors include tumor mutational load, PD-1 expression, CD4/CD8 lymphocyte ratio, the percentage of tumor-infiltrating lymphocytes, and several methods of establishing immune scores [1,2,3].

However, selecting patients by basic characteristics remains an acceptable alternative, but the efficacy of such a selection still must be demonstrated. The obesity paradox, for example, is an interesting concept recently described for immunotherapy. It seems that overweight and obese patients have a better response to nivolumab when compared to normal-weight patients [4,5]. Cachexia and sarcopenia, on the other hand, are negative prognostic factors [6,7].

The central idea of the present paper is to establish whether sarcopenia, indirectly measured through creatinine levels or nutritional status may serve as predictive factors for nivolumab efficacy. All in all, it seems that response to immunotherapy is more feasible before neoplastic disease consumes most of the system resources.

## 2. Results

A total of 57 (n = 57) patients were included in the trial. The characteristics of the patients were described in Table 1. The male:female ratio was 44:13, with a median age of 62 (standard deviation of 10.87), mean baseline creatinine value of 0.93 (standard deviation of 0.23), mean baseline BMI of 24.6 (standard deviation of 4.87), and a mean follow-up time of 12 months. Primary tumor distribution was seven patients with renal carcinoma (12.28%), 26 with melanoma (45.61%), and 24 with NSCLC (42.1%). Only 8.77% of the patients had an Eastern Cooperative Oncology Group (ECOG) performance score of 2, while all others were in good clinical condition, with ECOG 0 or 1. The proportion of underweight/normal weight/overweight–obese patients was: n = 6 (10.52%) were underweight, n = 26 (45.61%) were normal weight, and n = 25 (43.85%) were overweight/obese. The best response distribution included 18 patients (31.6%) with stationary disease at three months, 22 (38.6%) with partial response, and 17 (29.8%) with progressive disease. There were no notable variations in the creatinine levels when comparing baseline and three months later.

The overall response rate (ORR) for the entire population was 38.59% (*p* = 0.02). Patients with BMI lower than 25 had an ORR of 28.5% (*p* = 0.003), whereas patients with BMI higher than 25 had an ORR of 42.3% (*p* = 0.002). Normal BMI patients were the most prone to weight gain, 8 out of 26 patients (30.7%). Patients with weight gain between the first and the follow-up assessment had the best ORR of 51.2% (*p* = 0.0042), closely followed by the patients with creatinine values higher than 0.9 mg/dL, where the ORR was 48.9% (*p* = 0.001). Progression-free survival was also superior in these two groups, so patients who gained weight had a lower probability of progressive disease (OR = 0.4 [95% CI; 0.4–1.2]), as did patients with creatinine higher than 0.9 (OR = 0.39 [95% CI: 0.13–1.14]).

**Figure 1:**
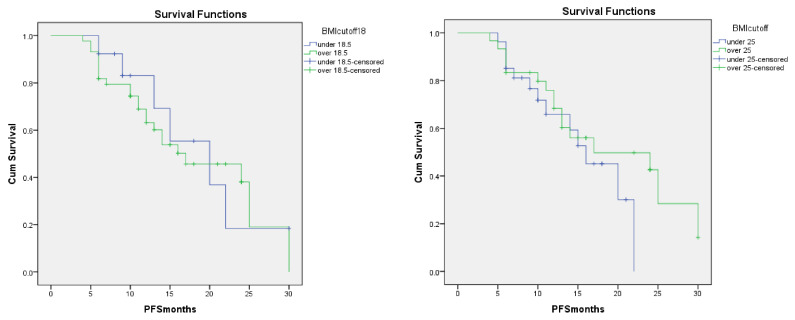
Comparison of PFS for the two separate groups, i.e., when using the BMI cut-off value 18.5 vs. 25.

No superiority was found in progression free survival when patients were dichotomized for BMI = 25 or BMI = 18.5 (Figure 1). Mean PFS for the BMI under 18.5 group was 10.2 months [95% CI: 5.8–23.1], versus 11.2 for BMI over 18.5 [95% CI: 5.3–25.3], *p* < 0.03. Mean PFS for the BMI under 25 was 11.2 months [95% CI: 7.2–20.1] vs. 13.3 months [95% CI: 6.4–22] for BMI over 25, *p* < 0.001.

**Figure 2:**
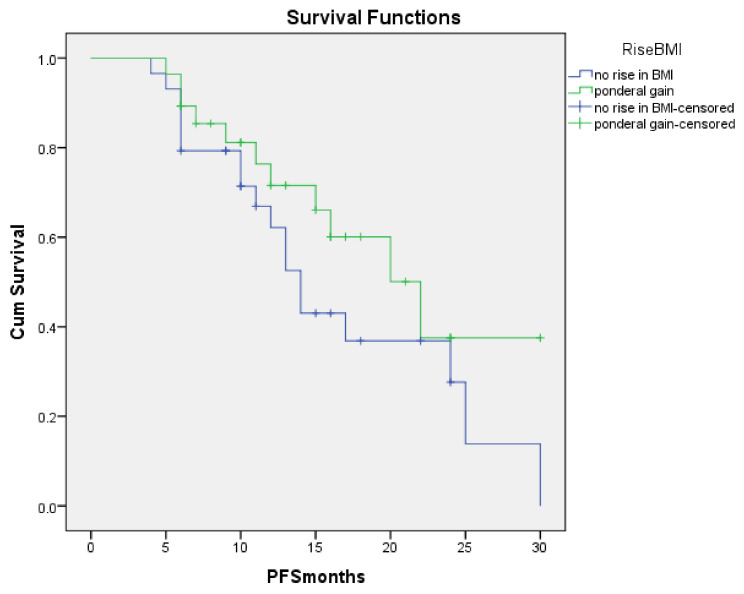
PFS for patients who had weight gain vs. patients who did not after three months of nivolumab treatment.

PFS rates for patients who had weight gain vs. patients who did not are represented in Figure 2. We found a significant difference between the two groups, where the ones with no rise in BMI at three months after treatment initiation, the mean PFS was 16.4 months [95% CI: 12.16–17.9] vs. 20.1 months [95% CI: 16.1–24.1] for those who had weight gain, *p* = 0.078.

**Figure 3:**
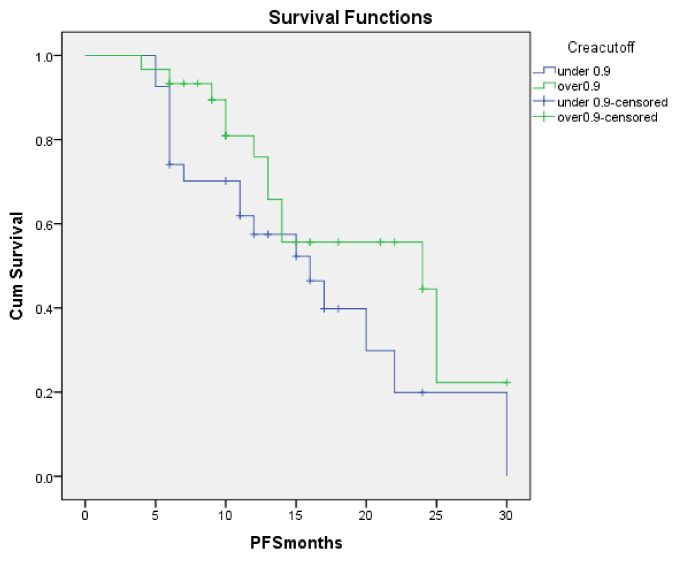
PFS for patients with creatinine under 0.9 vs. creatinine over 0.9 (baseline values).

There were also differences in PFS in the patients with baseline creatinine over 0.9 when compared with under 0.9 values as Figure 3 shows. Mean PFS in the first group was 19.78 months [95% CI: 16.23–22.9] vs. 16.1 [95% CI: 12.2–20.3], *p* < 0.001.

## 3. Discussion

The present paper demonstrated that the most valuable predictive factors for response to nivolumab are creatinine values higher than 0.9 and a rise in BMI during the first three months of treatment. BMI represents a biased modality of estimating adiposity because it does not give information on muscle/adipose tissue ratio, the most important aspect in our research. Therefore, it was considered that analyzing the two aspects simultaneously would give a better perspective on the patients’ profile.

Creatinine was chosen as a decisive variable in our analysis because it is considered a surrogate marker for sarcopenia. The loss of skeletal muscle mass is an indirect effect of the high catabolic effect of advanced cancer. However, in order for creatinine to illustrate this effect, no kidney dysfunction should be present; none of the enrolled patients had any history of such disease. The fact that female or older patients (age older than 65) have default lower creatinine levels was also taken into consideration when choosing the creatinine cut-off value [8,9].

Creatinine values indirectly related to sarcopenia are subject to debate. The literature proposes both 0.7 mg/dL and 0.9 mg/dL values [4]. The authors considered a higher value more reliable because it lowered the importance of gender-based value differences and that of the age differences. The gender differences focus on different muscle metabolism patterns and are considered to be one of the reasons males have better response rates to immunotherapy than females [10,11].

It was demonstrated that patients with lower muscle mass had weaker immune responses. This is mainly due to the relationship between muscle proteins and leucocyte function [12]. Glutamine, for example, if insufficient, can alter monocyte function by lowering levels of adenosine triphosphate. When this happens, the expression of surface cellular markers is inadequate and phagocytosis is inefficient. There is clear proof that sarcopenia leads to worse outcomes in sepsis [12,13].

In the present analysis, no superiority in PFS was found when stratifying either for BMI = 18.5 or BMI = 25, but the largest proportion of patients were overweight or obese. The capacity of the analysis to evaluate the PFS differences was also lower due to the uneven number of female vs. male patients.

Inequity in weight groups was reported in other studies, too. The predominance of overweight and obese patients in patients with melanoma or NSCLC was usually described in the literature, with around 60% being reported in this category.

The hypothesis that weight gain is a positive prognostic and predictive factor is not new either, with the same concept demonstrated in relation to chemotherapy. This was demonstrated for some other tumors as well [14,15,16].

A linear correlation was found in a retrospective trial between BMI rise and outcome (both PFS and overall survival) in melanoma patients treated with either checkpoint inhibitors or targeted therapy. This benefit was maintained even in morbidly obese patients [5].

The fact that all the patients were treated with fixed doses of nivolumab excludes the possibility that the different outcomes were explained by dosage per kg; the authors consider this a positive aspect of the present analysis.

The connection between adipose tissue and immune processes was described in recent literature, and it is well known that fatty tissue can help maintain immune response. Some of the processes that explain this link are the activation of T regulatory cells through adiponectin, maintaining the balance between Th1 and Th2 cells, and the implication of the adipose cell in the CD20 pathway [17,18].

Some argue that adipose tissue can serve as a pool of memory T cells by offering them a useful microenvironment after an infectious process, for example. Even if these cells can accumulate, there is no certainty that they remain efficient after a period of inactivity [19].

One trial that included obese mice demonstrated that exhausted T reg cells were present in large numbers in these populations. Paradoxically, these T reg cells can be brought back to activity by stimulating the pathway that includes the PD-1 receptor, the exact target of nivolumab [19]. This is one possible explanation of the so-called “obesity paradox” observed with immune checkpoint inhibitors.

## 4. Materials and Methods

The present trial is an observational, retrospective one that included patients who received nivolumab for metastatic melanoma, renal carcinoma or NSCLC between 2018 and 2020 in 3 centers in Romania: Elias University Emergency Hospital in Bucharest, County Clinical Emergency Hospital of Constanta, and Al. Trestioreanu Institute of Oncology in Bucharest. The present paper gained approval of the Ethics Committee of Elias University Emergency Hospital, reference no. 1/26th May 2020.

Prior kidney disease was an exclusion criterion. The trial focused on creatinine levels both at treatment initiation and at first follow-up (at three months) and on the BMI (body mass index) of the patients for the same intervals, as recorded in the patients’ charts. BMI was calculated with the following dedicated formula:

*BMI = Weight (kg)/(Height (m))*^*2*^ (1)

Patients who had a BMI lower than 18.5 were classified as underweight, between 18.5 and 24.9 as normal and over 25 as overweight and obese. Then, they were dichotomized into BMI under/over 18.5 and under/over 25. A cut-off for the levels of creatinine was established at 0.9 mg/dL and the levels were recorded at the same intervals as for BMI. Progression-free survival was the primary endpoint of the trial, and this was defined as time to lack of clinical benefit or death, whichever came first. BMI and creatinine were the primary variables by which progression-free survival (PFS) was assessed and compared. The Kaplan–Meier method was used for this purpose; the *p*-value was calculated using log-rank test.

Other used variables included age, gender, ECOG performance status, treatment line, and best response (defined as stationary disease by iRECIST (immune response evaluation criteria in solid tumors) on first follow-up imaging, partial response, or progressive disease). All patients were radiologically evaluated after 3 or 4 months of nivolumab treatment. ORRs (overall response rates) were established after consulting these imagistic reports. ORR was defined as the percentage of patients who had any reduction in tumor volume during the follow-up time. To establish whether the patient had progressive disease or not, the treating physician gathered and analyzed all the information (clinical condition, imaging studies). Progressive disease was decided in a multidisciplinary board. Data regarding these variables were collected from the patients’ individual reports. All patients received fixed doses of treatment, either nivolumab 240 mg every 2 weeks or 480 mg every 4 weeks (in the case of some melanoma patients). Statistical analysis was done using IMB SPSS version 20.

## 5. Conclusions

This trial demonstrated that patients treated with nivolumab who have weight gain during treatment have better PFS than those who do not. Creatinine levels of over 0.9 at treatment initiation can also have positive predictive value. These findings would be very easy to follow in daily clinical activity, but they need validation in larger, prospective trials.

## Figures and Tables

**Table 1:** Characteristics of the patients.

Characteristic	N (%)
Female	13 (22.8%)
Male	44 (77.19%)
Age	
Mean	62
Range	[39–80]
Site of tumor	
Melanoma	26 (45.61%)
NSCLC	24 (42.1%)
Renal cell carcinoma	7 (12.28%)
Proportion of weight groups	
Underweight	6 (10.52%)
Normal	26 (45.61%)
Overweight/obese	25 (43.85%)
ECOG	
ECOG = 2	5 (8.77%)
ECOG = 1	12 (21.05%)
ECOG = 0	40 (70.17%)
Creatinine	
Mean creatinine level [Range]	0.93 [0.52–1.74]
Creatinine over 0.9	27 (47.4%)
Creatinine under 0.9	30 (52.6%)
BMI values	
BMI under 18.5	6 (10.6%)
BMI over 18.5	51 (89.4%
BMI under 25	27 (47.4%)
BMI over 25	30 (52.6%)
